# Pamphlets Received

**Published:** 1855-07

**Authors:** 


### *Pamphlets Received.*

*Cases of Polypus of the Womb.* With Remarks by WALTER CHANNING, M. D. Republished from the Boston Medical and Surgical Journal.

This is a collection of the results of the author's experience. He has seen twenty-two cases, and operated in sixteen—a very unusual number of cases for a single practitioner. Two instances occurred in which the polypus returned, but Dr. Channing thinks that it does not return from the same spot. This was proved by autopsy in one of the cases. In cases of what he calls *concealed* polypus, he advises ergot at the catamenial period to bring it within reach. We quote a sentence: "Ergot can here do no harm. On the contrary, it often tends directly to check uterine and *other* hæmorrhages."

The emphasis given to the word *other*, leads us to suppose that Dr. Channing may have used ergot, as we saw it used a week since by a friend in the country, to check hæmoptysis. Will not our friend, or some one else, "post us up" on this new use of ergot?

*On the Nature of Malaria, and Prevention of its Morbid Agency.* By JOHN GORRIE, M. D., of Apalachicola, Florida.

This is a reprint of an article from the New Orleans Medical and Surgical Journal. The new hypothesis as to the nature of malaria is, that it is a product of decomposition analogous to the volatile oils, and numerous resemblances and identities between its known properties and those of these oils are mentioned.

Dr. Gorrie's means of prevention, however, seems to have reference entirely to the humid element of malaria, and though he does not mention the dew-point theory of malarial activity, he virtually concedes it. By a proper construction of a house he would have áll air (in summer) introduced from above, and passed over a receptacle containing ice. Dr. C. assumes that the air by this means would be deprived of organic elements, and undergo an electrical change, but it is very evident that its principal effect would be to deprive the warm current of most of its moisture, and insure a low dew-point in the room.

*Observations on Wounds of the Heart, and their Relations to Forensic Medicine, with a Table of forty-two recorded cases.* By SAMUEL S. PURPLE, M. D.

This is a valuable statistical paper, especially important in legal medicine. Dr. Purple arrives at the conclusion that wounds of the heart are not usually immediately fatal, that recovery is possible, and judicious treatment important; that a ball may be imbedded in the walls of the heart without precluding recovery; that incised wounds of the heart may heal by first intention; that life may continue for days with a ball in a heart cavity; that the diagnosis of wounds of the heart is very uncertain; and that their prognosis is unfavorable, but not hopeless.

